# Bridging Locomotion and Manipulation Using Reconfigurable Robotic Limbs via Reinforcement Learning

**DOI:** 10.3390/biomimetics8040364

**Published:** 2023-08-14

**Authors:** Haoran Sun, Linhan Yang, Yuping Gu, Jia Pan, Fang Wan, Chaoyang Song

**Affiliations:** 1 Department of Mechanical and Energy Engineering, Southern University of Science and Technology, Shenzhen 518055, China; 12150050@mail.sustech.edu.cn (H.S.); 11950013@mail.sustech.edu.cn (L.Y.); 11611307@mail.sustech.edu.cn (Y.G.); 2 Department of Computer Science, The University of Hong Kong, Hong Kong SAR, China; 3 Shenzhen Key Laboratory of Flexible Manufacturing and Robotics, Southern University of Science and Technology, Shenzhen 518055, China; 4 School of Design, Southern University of Science and Technology, Shenzhen 518055, China; 5 Guangdong Provincial Key Laboratory of Human-Augmentation and Rehabilitation Robotics in Universities, Southern University of Science and Technology, Shenzhen 518055, China

**Keywords:** loco-manipulation, reinforcement learning, reconfigurable robot

## Abstract

Locomotion and manipulation are two essential skills in robotics but are often divided or decoupled into two separate problems. It is widely accepted that the topological duality between multi-legged locomotion and multi-fingered manipulation shares an intrinsic model. However, a lack of research remains to identify the data-driven evidence for further research. This paper explores a unified formulation of the loco-manipulation problem using reinforcement learning (RL) by reconfiguring robotic limbs with an overconstrained design into multi-legged and multi-fingered robots. Such design reconfiguration allows for adopting a co-training architecture for reinforcement learning towards a unified loco-manipulation policy. As a result, we find data-driven evidence to support the transferability between locomotion and manipulation skills using a single RL policy with a multilayer perceptron or graph neural network. We also demonstrate the Sim2Real transfer of the learned loco-manipulation skills in a robotic prototype. This work expands the knowledge frontiers on loco-manipulation transferability with learning-based evidence applied in a novel platform with overconstrained robotic limbs.

## 1. Introduction

Loco-manipulation is an essential skill for intelligent agents, combining locomotion and manipulation. We have seen humans and animals frequently switch limbs to perform different functions. Skilled circus acrobats can manipulate large, heavy objects using their legs with their backs facing the ground. Dung beetles roll a dung ball with their four rear legs as if they were walking on it [1]. Seals or quadrupedal animals usually use their limbs for swimming or locomotive traversal across complex terrains and can even manipulate objects with their limbs after acrobatic training, inspiring robots to learn [2]. Observations from nature indicate a duality between locomotion and manipulation: locomotion can be viewed as manipulating oneself around the environment [3,4] or manipulating the supported terrain [5], making it an interesting research question to explore the loco-manipulation problem regarding the transferability between these two skills, where a combination of bio-inspired design and robot learning presents as a promising method for further investigation.

Reinforcement learning has been proven effective in learning robotic control [6,7,8], as well as in agile locomotion [9,10,11,12] and in dexterous manipulation [13,14]. A typical design of loco-manipulation involves adding a manipulator to a legged robot [15,16], where a whole-body control strategy could be implemented to learn a unified policy for manipulation during locomotion [17]. However, due to the topological difference between the quadrupedal base and the manipulator attached, skills cannot be shared between the upper and lower limbs [18]. Another way of achieving loco-manipulation is to use some or all robotic legs to manipulate objects [2,19,20,21,22]. Recent work presented a goalkeeper quadruped that manipulates a soccer ball using its legs [21]. Locomotion and manipulation skills are trained separately in [22] and integrated using a behavior tree to achieve long-horizon loco-manipulation tasks such as climbing a wall and pressing a button. These studies successfully learned locomotion and manipulation skills on a single robot, with the robotic limbs functioning as legs and fingers. Researchers achieved in-hand manipulation of a ball by viewing the four legs of a quadruped robot as four fingers of a dexterous hand [2]. However, the low-level policies for reinforcement learning of manipulation and locomotion are still separated, and the skill-level relationship between locomotion and manipulation has yet to be explored in the current literature.

While multilayer perceptron (MLP) models are widely adopted in building reinforcement learning models for efficient feature extraction from the data, recent development in graph neural networks (GNN) offers a different approach that considers the reactive and sensory architecture of the robotic topology for learning skills such as locomotion and manipulation [23,24]. For example, GNN-based RL policies explicitly parameterize the interaction between entities (i.e., joints or links) with neural networks. They have made considerable progress in exploiting the structural information in policy learning and have shown the potential for generalization of different configurations experimentally [25,26]. These models perform significantly better on structured transfer learning tasks and even in a zero-shot setting. Learning a structured policy with GNN shows excellent potential to unify the loco-manipulation training, which complements the MLP-based reinforcement learning for loco-manipulation.

The design of the robotic platform also plays a critical role in materializing transferable loco-manipulation skills, in addition to the algorithmic computation in learning-based methods. For object manipulation by hand and omni-directional locomotion on land, the design challenge involves differentiated workspaces, task requirements, and object-centric interaction. i.e., most grippers for dexterous manipulation are much smaller than quadrupedal robots for walking on the ground, making it challenging to reuse the existing platform for both tasks directly. The ROBEL platform is an example built by low-cost servo motors as robotic limbs. It is suitable in size for both tasks but is limited by the serial open chain design for the robotic limb [27]. The TriFinger platform is a recent attempt that leverages the quasi-direct drive (QDD) design for robotic legs to build a three-fingered platform for object manipulation [28]. Through open-source design and international competitions, these works raise awareness of a learning-based solution to loco-manipulation problems with design [29]. For comparative analysis, it would be preferred to test these two skills, or the transferability between them, using a unified, representative, yet reconfigurable platform that signifies the design features of the multi-limbed robot for locomotion and manipulation.

With the design of reconfigurable robots, this paper aims to bridge locomotion and manipulation by investigating their connections using reinforcement learning. Our contributions are: (1) we propose a loco-manipulation formulation to unify locomotion and manipulation tasks and develop reconfigurable robots using overconstrained robotic limbs; (2) we transfer learned policies between locomotion and manipulation and analyze the transferability between the two skills in two robot configurations; and (3) we learn a unified policy for loco-manipulation tasks with MLP and GNN, providing data-driven evidence supporting locomotion and manipulation’s duality.

The paper is organized as follows. In Section 2, we present the unified loco-manipulation models, methods used to train loco-manipulation skills, and the experimental setups. In Section 3, we report and analyze the results from the experiments. Section 4 concludes this paper with a discussion of the limitations and future work.

## 2. Materials and Methods

### 2.1. Problem Formulation

Despite the difference between locomotion and manipulation problems, robot-environment interactions for the two tasks have a lot of similarities. Taking a robot-centric view, locomotion and manipulation problems can be unified since, in both situations, the robot exerts forces to change the state of its surroundings using its limbs. Starting from this idea, we unify the formulation of locomotion and manipulation problems by viewing locomotion as the manipulation of the supported terrain “object,” as pointed out in [5]. For example, the quadruped robot rotating clockwise in place relative to the terrain is equivalent to saying that, from the robot’s view, the ground floor is “rotating” counter-clockwise relative to the robot itself. The “rotation” of the ground floor is the same as when a multi-fingered robot is rotating an object counter-clockwise. This unified loco-manipulation model makes transferring policies between locomotion and manipulation tasks possible. A unified loco-manipulation policy can also be learned using MLP and GNN, which naturally resembles the loco-manipulation model and provides better interpretability to the actual physical robot–environment interactions in loco-manipulation tasks.

We formulate the locomotion and manipulation problems as an infinite-horizon discounted Markov decision process (MDP) [30]. To fully describe the MDP for the continuous control problems, we define the observation space as *S* and the action space as *A*. To interact with the environment, the agent generates a stochastic policy $\pi_{\theta }\left(a_{t}|s_{t}\right)$ based on the current state $s_{t}\in S$, where $a_{t}\in A$ is the action and θ signifies the parameters of the policy function. Then, the environment produces a reward $r(s_{t},a_{t})$ for the agent, and the agent’s objective is to find a policy that maximizes the expected return.

### 2.2. Towards a Unified Loco-Manipulation Model

**Figure 1:**
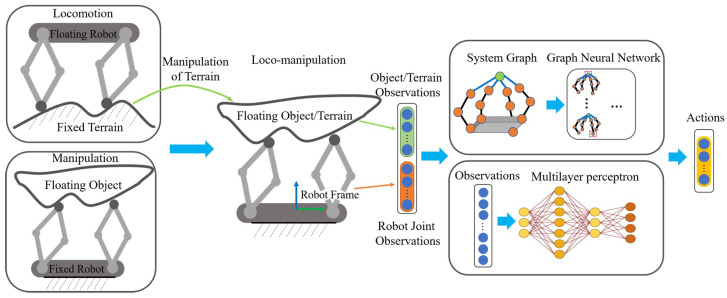
Locomotion as manipulation of terrain to unify the formulation of loco-manipulation problems, providing a unified observation as input to the GNN and MLP models.

Legged robots are often modeled to have a free-floating base relative to the fixed terrain. In contrast, multi-fingered robotic hands generally have a fixed base, such as in [14], with objects free to move in space. The conventional modeling method has hindered the unification of the observation space, leading to different agents’ policies for the two problems. Despite the difference between the dynamics of the locomotion and manipulation problems [4], a unified formulation is still possible given the observation that the motion of the multi-legged robot is the opposite movement of the terrain relative to the robot base [5]. As illustrated in Figure 1, we view the terrain in locomotion problems as the object to be manipulated.

Thus, the locomotion of the multi-legged robot becomes the manipulation of the terrain [5]. The states of the robot base are transformed into the states of the terrain “object” expressed in the robot base frame using coordination transformation and Newtonian relativity.

Specifically, for the locomotion problems, we define the transformation to be:

(1) $$s^{\prime }=T\left(s\right)=-R_{RT}\cdot s,$$

where $s^{\prime }$ refers to the state of the terrain “object”, such as position, linear/angular velocity, or orientation expressed as rotation matrix. $R_{RT}$ is the rotation matrix from the robot base to the terrain frame, and *s* refers to the state of the robot base expressed in the terrain frame. With the transformation, the robot–environment interaction for manipulation and locomotion can be unified as a generalized loco-manipulation model with a fixed robot base and a free-floating object or terrain.

### 2.3. Learning Algorithms

**Task Description**: Our unified loco-manipulation formulation can be generalized to other locomotion and manipulation tasks, such as walking toward a target or manipulating objects with various shapes. This paper focuses on one pair of locomotion and manipulation tasks. The goal of the locomotion task is to control the quadruped robot on flat terrain so that the orientation of the robot base matches the target rotation without a large translation. As explained above, the task is equivalent to the control of the terrain “object”. A robot with the same topological configuration as the quadruped robot is inverted upside-down to become a four-fingered manipulator, as shown in Figure 1. The goal of the manipulation task is to control the orientation of a flat plate object to match the goal rotation.

The observation space is defined as $S=(p,v,\omega ,n,\Delta q,\theta ,\dot{\theta },a,a^{\prime })$, where *p*, *v*, and ω are the positions, linear velocity, and angular velocity of the terrain or the object expressed in the robot base frame, respectively; *n* refers to the unit normal vector of the flat terrain or plate object, which is similar to the gravity vector used in [11]; Δq is the quaternion difference calculated from $q\cdot q_{goal}^{*}$, with *q* being the quaternion of the object (flat terrain or plate object) and $q_{goal}^{*}$ being the conjugate of the goal quaternion; θ and $\dot{\theta }$ are joint positions and velocities; and *a* and $a^{\prime }$ are current and last actions, respectively. Action space $a=(a_{1},a_{2},\ldots ,a_{12})$ denotes the goal velocity command for each joint.

The reward function contains dense and sparse rewards $r\left(s_{t},a_{t}\right)=r_{d}+r_{s}$. Dense reward $r_{d}$ consists of rotation reward 1/(0.1+Δθ) to drive the robot to the goal rotation, position deviation penalty −||p|| to encourage the robot to stick to the original position, joint acceleration penalty $\left|\right|\dot{\theta }-{\dot{\theta }}^{\prime }\left|\right|$ to punish the sudden moves of robot joints, and action rate penalty $||a-a^{\prime }\left|\right|$ to punish significant joint command changes. The above reward terms are multiplied by specific reward scales at each time step, as shown in Table 1. The agent is also given a sizable sparse reward $r_{s}$ upon success. To encourage the agent to control the orientation of the object robustly, we define success to be the angle difference $\Delta \theta =2\sin^{-1}(||q\cdot q_{goal}^{*}\left||\right)$ within a threshold value $\bar{\theta }$ for 15 simulation steps (0.5 s) consecutively. In this paper, we set the threshold to 0.15 rad. The goal rotation is randomly sampled from a fixed range of Euler angles, with roll and pitch from −0.4 to 0.4 rad and yaw angle from −1.57 to 1.57 rad. The environment terminates whenever the robot self-collides, any part except its tips (feet or fingertips) touches the object or terrain, or the agents fail to succeed within 10 s.

**Model Architecture and Learning Details**: We use MLPs as our policy and value models. They share most of the parameters except for the last layer. In the last layer, the policy model receives a 12-dimensional output as the action for each joint, and the value model outputs the predicted state value used in policy updates. We use Proximal Policy Optimization (PPO) [31] to optimize the agent’s cumulative return. PPO is an on-policy reinforcement learning algorithm where the agent switches between interacting with the simulated environment to collect trajectories and optimizing surrogate objectives utilizing the sampled trajectories. The Adam optimizer is used to adjust the learning rate. Hyperparameters of PPO for MLP policies can be found in Table 2.

### 2.4. Learning a Structured Policy with Inductive Bias

Furthermore, we exploit a structured policy for loco-manipulation control problems while considering prior structural information. The structural information is explicitly represented by an undirected graph. Since these two systems share a similar topological structure, locomotion and manipulation are represented in the same graph, as shown in Figure 1.

We use the neural network to represent the interaction between different nodes: object node and joint node, as well as joint node and joint node. The network propagates information over the structure of the agent and then predicts actions for each joint of the agent. We aim to exploit the physical structure and physical dependencies that naturally exist in loco-manipulation systems.

**Graph Construction**: Our approach relies on the fact that bodies of robot–object systems have a discrete graph structure. This system is represented by a graph where nodes of the graph correspond to the joints and edges correspond to the physical dependencies between them. There are two types of nodes: twelve robot joint nodes and one object node. The object node features $V_{O}$ consist of the position, linear and angular velocities, unit normal vector, and quaternion difference of the terrain or the object expressed in the robot base frame. The robot node features $V_{R}$ consist of joint positions, velocities, current, and the last action command for each joint. We construct the edges based on the physical dependencies.

**Encoder**: The model receives a system graph in each time step, containing the object node, robot node, and edges, as $G=(V_{O},V_{R},E)$. The encoder maps the node features to a fixed-dimension embedding as follows:

(2) $$h_{r}=\sigma_{r}\left(v_{r}\right),h_{o}=\sigma_{o}\left(v_{o}\right),$$

where σ is an MLP in this paper. This encoder contains two separate MLPs, denoted as $\sigma_{r}$ and $\sigma_{o}$.

**Message Propagation**: The propagation model consists of *N* stacked graph network blocks [32] to update node embedding. In each graph network, the model first computes the message between each connected node: $m_{o,r_{i}}=\gamma_{or}\left(h_{o},h_{r_{i}}\right)$ for object node and robot node; and $m_{r_{i},r_{j}}=\gamma_{rr}\left(h_{r_{i}},h_{r_{j}}\right)$ for two robot nodes, where γ denotes the MLP and *h* denotes the feature embedding. Note that all edges of the same edge type share the same instance of the message function.

Once every node finishes computing messages, messages are aggregated from all incoming neighbors of each node: $h_{i}=\max(\{m_{v_{i},v_{j}}\left|\right|v_{j}\in C\left(v_{i}\right)\},\;dim=1)$, where $v_{i},v_{j}$ denote the connected nodes containing the robot node and the object node, and $C(v_{i})$ denotes all nodes connected to the node $v_{i}$.

This message computation and aggregation are recurrently applied *K* times in each graph network block, resulting in N×K updates. Additionally, we found that using shareable parameters for computation models of different edges did not reduce the experimental performance. Therefore, we adapted the shareable parameters to increase computing efficiency.

**Decoder**: The decoder model consists of two MLPs corresponding to the policy and value models in reinforcement learning. They share the encoder and propagation computation parameters. For the policy model, the output should be the mean value of a Gaussian distribution for each joint action: $a_{r}=o_{r}\left(v_{r}\right)\in R^{1}$, where $v_{r}$ denotes the joint node. For the value model, we first use a graph-level pooling $H\in R^{N\times D}\to \hat{H}\in R^{D}$, where *H* denotes the feature matrix for *N* nodes and predicts the state value using $V=o_{v}\left(\hat{H}\right)\in R^{1}$. Hyperparameters of PPO for GNN policies are listed in Table 3.

### 2.5. Reconfiguration with Overconstrained Robotic Limbs

**Figure 2:**
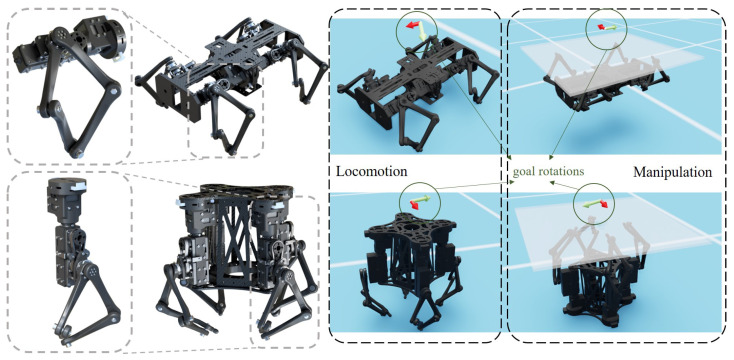
Horizontal robot configuration (**top**) and vertical robot configuration (**bottom**). Locomotion tasks in the simulation are to control the rotation of the robot base; manipulation tasks are to control the rotation of the translucent plate. The frame icons on top of the robots indicate the current goal rotations of the robot base or the plate.

We adopted the design of an overconstrained robotic limb [33,34,35] module described in [36] and added a hip motor to each limb. Each module consists of three actuated revolute joints, with two coaxial ones driving the overconstrained linkages. Depending on the orientation of the limb module relative to the robot base, two robot configurations are designed with four robotic limbs, namely the horizontal and vertical configurations, as shown in Figure 2.

Both designs can be reconfigured as quadruped for locomotion and a four-fingered gripper for manipulation (with an inverted base, which can be attached to a manipulator but considered fixed in the following analysis). Robots are weighted about 2.5 kg for both configurations. The terrain is assumed to be flat, and the object in the manipulation task is a plate weighing around 2.4 kg, with the dimensions 50 cm × 50 cm × 0.8 cm.

### 2.6. Simulation Environment

Isaac Sim was used to simulate locomotion and manipulation tasks as it supports vectorized simulation, making a massively parallel training regime possible to accelerate reinforcement learning. For all experiments, 2048 isolated and parallel environments were created, and each environment consisted of a robot and a plate object (for manipulation tasks) or fixed flat terrain (for locomotion tasks). In the simulation, maximum output torque and velocity were limited to 1.5 Nm and 3 rad/s, respectively, consistent with the actual performance of the physical servo motors. The robot joints were velocity controllers, and the actions of the policies were goal joint velocity commands from −3 to 3 rad/s. We used PPO and MLP implementation in SKRL  [37] and implemented our graph model to train the structured policies. The following experiments were conducted for both horizontal and vertical configurations:

- **Learning MLP Policy from Scratch**: We trained agents using the MLP policy from scratch. The locomotion task (LocoFromScratch) and manipulation task (ManiFromScratch) were trained in separate simulation runs, as shown in Figure 2.
- **Learning MLP Policy Transferred from Expert Policy**: The policy learned from scratch was transferred to another task. Specifically, for the locomotion transfer experiments (LocoFromMani), the expert manipulation policy trained from the ManiFromScratch experiment was used as the initial policy, while for the manipulation transfer experiments (ManiFromLoco), the initial policy was set to the policy trained from LocoFromScratch.
- **Co-learning a Unified Loco-manipulation Policy**: We combined the locomotion and manipulation task into a single simulation run and trained a unified policy using MLP policy (ColearnMLP) or graph policy (ColearnGraph).

All MLP policies contain three hidden layers with 256, 128, and 64 neurons, respectively. GNN consists of three stacked blocks. The message computation and aggregation are recurrently applied twice in each block, with 32 neurons in each layer.

## 3. Experiment Results

### 3.1. Transferability between Locomotion and Manipulation in Two Robot Configurations

We tested the zero-shot transferability between the two tasks. We randomly generated 2048 goal rotations and evaluated the success rate of the locomotion task using an expert manipulation policy or the manipulation task using an expert locomotion policy without further training. As shown in Table 4, we observed a noticeable transferability between locomotion and manipulation for the horizontal robot configuration. However, the transfer success rate for the vertical robot configuration was comparatively much lower.

**Figure 3:**
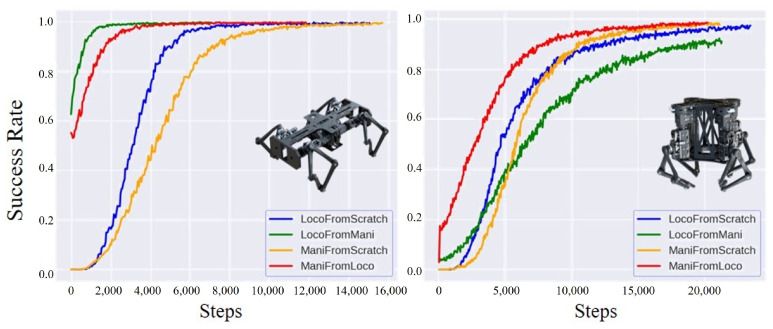
Performance of loco-manipulation transfer learning for the horizontal configuration (**left**), and the vertical configuration (**right**).

We continued to train the tasks transferred from the expert policy. We found a high correlation between zero-shot transferability and the learning curve of the success rates, as shown in Figure 3. For the horizontal robot configuration, which had a high transferability between two tasks, the success rate converged much faster when transferring from another task than learning from scratch. For vertical configuration, the manipulation task benefited from the locomotion policy, but we observed a negative impact of the manipulation policy on learning the locomotion task.

From the above results, we found different transferability for different robot configurations. Zero-shot transferability was higher for the horizontal configuration compared to the vertical configuration. Additionally, the success rates of the unified loco-manipulation policy converged much slower for the vertical configuration than for the horizontal configuration. The horizontal robot configuration generally had better transferability between locomotion tasks and manipulation tasks. The lower transferability of the vertical configuration might be because of the robot’s higher center of mass and the inward bending characteristic of the overconstrained limb [36]. The effective workspace of the limb module is smaller than the horizontal configuration, which makes the robot and the plate prone to falling. This difference indicates that the hardware design is essential and substantially impacts transfer learning between locomotion and manipulation tasks.

We also found that transfer learning caused behavioral differences. We randomly generated five goal rotations and evaluated the policies of LocoFromScratch, LocoFromMani, ManiFromScratch, and ManiFromLoco of the horizontal configuration. Joint position trajectories during each evaluation were recorded. We calculated the average joint position difference to investigate the influence of transfer learning on the behavior change of the robot doing locomotion or manipulation tasks. For locomotion, we calculated the average joint position difference between LocoFromMani and LocoFromScratch. Additionally, the difference between LocoFromMani and ManiFromScratch is also calculated in Table 5. Results show that the behavior of locomotion transferred from the manipulation policy is closer to that of manipulation learned from scratch. This means that some manipulation skills are *inherited* in the locomotion task, making the training of locomotion task transferred from expert manipulation policy take a different route from training locomotion from scratch. This behavioral *inheritance* also applies to the manipulation task transferred from the locomotion policy.

### 3.2. Co-Learning Unified Loco-Manipulation Policies with MLP and GNN

**Figure 4:**
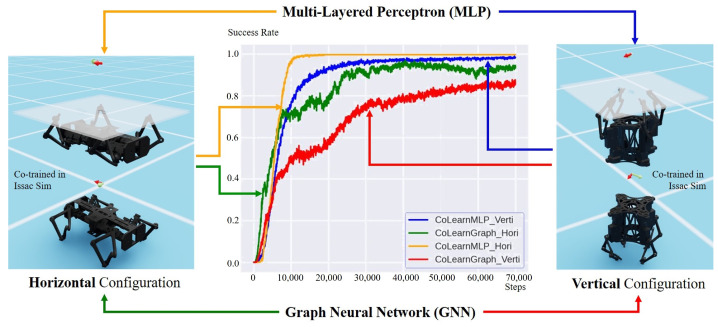
Performance of unified loco-manipulation learning (**middle**) for the horizontal configuration (**left**) and the vertical configuration (**right**).

We report the performance of unified loco-manipulation training, as shown in Figure 4. Although the results of zero-transfer learning in Table 4 indicate that there is a clear gap between manipulation and locomotion tasks, especially for the vertical configuration, our method could bridge this gap and find a unified policy for both, i.e., a single brain for both manipulation and locomotion control. MLP and GNN converge to a high success rate (more than 80), although GNN typically needs more data. Additionally, MLP achieves a better performance on the horizontal and vertical configurations.

One possible reason is that GNN tends to learn the general representation of the interaction between entities, such as objects and joints. This representation should be compatible with different entities since they all share the same parameters. To contrast, MLP parameterizes each pair of observations defined in this task. Moreover, careful hyperparameter (learning rate, reward function scale) fine-tuning is needed in training the GNN model to produce a comparable performance to MLP. However, the intrinsic modeling method of GNN could bring better explainability and extensibility to research further. The graph-based network can leverage the structural information encoded by the agent’s body, which is advantageous in learning the correct inductive bias. As a result, the GNN model tends to learn a smooth policy, and MLP performs more aggressively. Note that although we unify the formulation of locomotion and manipulation problems, we see a clear difference in behavior in these two tasks using a single policy, which exhibits the multi-task ability of our method.

### 3.3. Transfer Learned Skills to Physical Hardware

**Figure 5:**
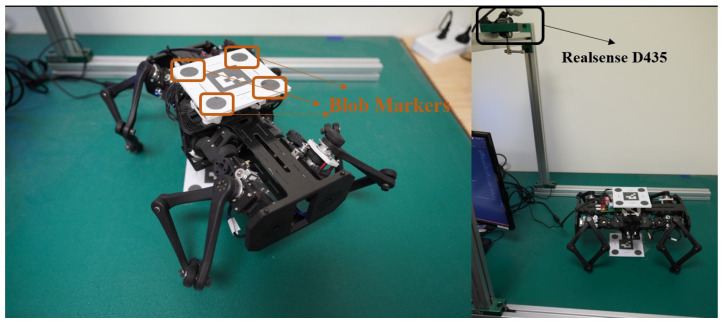
Robot hardware setup for the sim-to-real test of the horizontal robot configuration.

We also show that the skills learned in simulation can be transferred to the physical robot. To demonstrate the transferability of the locomotion skills to the physical robot, we set up an experimental platform as shown in Figure 5. The robot in the horizontal configuration comprises four overconstrained reconfigurable modules and 12 Dynamixel XM430-W270 servo motors. The marker board on top of the robot has four blobs, and we use ViSP [38] to track the pixel positions of the blobs in the camera frame of Realsense D435. The positions of the blobs are then used to estimate the 6D pose of the robot.

We investigated evidence of the sim-to-real transferability by replaying the joint position commands from the simulation to the physical robot. We found a higher discrepancy between the joint velocity controller in Isaac Sim and the actual joint velocity controller of the servo motors. Therefore, we switched to position PD controllers in simulated and actual motors and re-trained the tasks. We set the controller mode for servo motors to current-based position control, with the maximum current set to 1.5 A, roughly equivalent to the maximum torque of 1.5 Nm. The profile velocities were set to 3.0 rad/s to limit the maximum joint velocities of the servo motors. In simulation and reality, we controlled a single servo motor to follow a fixed joint position trajectory. We fine-tuned the gains in simulation to minimize the difference in the trajectory between the sim and actual using Bayesian optimization [39]. We applied domain randomization on the scale and mass of the robot base, joint frictions, and friction coefficients of the terrain and the plate object. We proved sim-to-real transferability by replaying the joint position commands from the simulation to the physical robot, and we showed that the replayed motion of the actual robot has a slight difference from the simulated one. Videos comparing the behavior of the simulated and real robots can be found in Supplementary Material.

## 4. Conclusions and Limitations

This paper proposed a method to transform the locomotion problem into manipulating the supported terrain to derive a unified formulation of locomotion and manipulation tasks. Using the unified formulation, we showed that skills can be successfully transferred between the two tasks through the proper design of the robot hardware. We also successfully learned a unified loco-manipulation policy by training locomotion and manipulation tasks in an integrated environment. We present the effectiveness of both MLP and GNN in learning this unified policy, with MLP having higher sample efficiency and GNN having better explainability and extensibility.

One limitation of our work is that we are still building the entire pipeline of sim-to-real transfer. Additionally, we only include one set of locomotion and manipulation tasks. Locomotion on flat terrain and manipulation of a plate object have similar contact modes and may not be general enough. Another limitation is that, even though we successfully trained a unified GNN policy, we do not yet exploit the advantages of GNN for loco-manipulation learning. GNN has the advantage of structure transfer learning and needs to be verified in further experiments.

We will continue improving our hardware setup to achieve direct sim-to-real transfer. We plan to use the proposed methods for other locomotion and manipulation tasks and to investigate the transferability between more complex quadruped walking skills and the manipulation of more challenging objects such as cubes. Tactile sensing for manipulation and ground reaction force sensors for locomotion are vital for more complex object geometry and challenging terrains [40]. GNN has shown great potential in integrating sensing and structural information since it explicitly exploits the physical dependencies in the robot system. In the future, we plan to undertake more experiments on various configurations to evaluate the transferability of the GNN model. For example, the quadruped locomotion policy should be easily transferred to hexapod locomotion since all joint interactions share the same parameters in GNN [26].

## Figures and Tables

**Table 1:** Reward scales for MLP and GNN policies.

Reward	Reward Scale of MLP	Reward Scale of GNN
Rotation Reward	0.5	2
Position Deviation Penalty	2.5	2.5
Joint Acceleration Penalty	0.0005	0.002
Action Rate Penalty	0.02	0.02

**Table 2:** Hyperparameters of PPO for MLP policies.

Hyperparameter	Value	Hyperparameter	Value	Hyperparameter	Value
learning_epochs	5	mini_batches	1	discount_factor	0.99
lambda	0.95	kl_threshold	0.012	grad_norm_clip	1.0
ratio_clip	0.2	value_clip	0.2	clip_predicted_values	True
value_loss_scale	1.0	rollouts	48	min_learning_rate	$1\times 10^{-6}$

**Table 3:** Hyperparameters of PPO for GNN policies.

Hyperparameter	Value	Hyperparameter	Value	Hyperparameter	Value
learning_epochs	5	mini_batches	1	discount_factor	0.99
lambda	0.95	kl_threshold	0.012	grad_norm_clip	1.0
ratio_clip	0.2	value_clip	0.2	clip_predicted_values	True
value_loss_scale	1.0	rollouts	48	min_learning_rate	$5\times 10^{-6}$
model_size	32	stack numbers N	3	iteration numbers K	2

**Table 4:** Success rates of zero-shot cross-task transfer for loco-manipulation with two robot configurations.

Model Transfer	Horizontal	Vertical
LocoFromMani	66.4%	3.6%
ManiFromLoco	51.8%	18.0%

**Table 5:** Average joint position differences (in rad) for horizontal configuration.

Model Transfer	LocoFromMani	ManiFromLoco
LocoFromScratch	0.2024	0.1063
ManiFromScratch	0.1007	0.1923

## Data Availability

All data related to this work are hosted on the following project website: https://github.com/bionicdl-sustech/LocoManipulationRL.

## Supplementary Materials

The following supporting information can be downloaded at: https://www.mdpi.com/article/10.3390/biomimetics8040364/s1, Video S1: Bridging Loco-Manipulation Skills via Reinformcement Learning.
